# Nociceptive and Histomorphometric Evaluation of Neural Mobilization in Experimental Injury of the Median Nerve

**DOI:** 10.1155/2013/476890

**Published:** 2013-07-11

**Authors:** Marieli Araujo Rossoni Marcioli, Josinéia Gresele Coradini, Regina Inês Kunz, Lucinéia de Fátima Chasko Ribeiro, Rose Meire Costa Brancalhão, Gladson Ricardo Flor Bertolini

**Affiliations:** Laboratory of Injuries Study and Physical Therapy Resources of the State University of Western Paraná (UNIOESTE), Universitária Street, 2069, Jd. Universitário, P.O. Box 711, 85819-110, Cascavel, PR, Brazil

## Abstract

The carpal tunnel syndrome is the most common peripheral neuropathy in the upper limb, but its treatment with conservative therapies such as neural mobilization (NM) is still controversial. The aim of this study was to investigate the efficacy of the NM as treatment in a model of median nerve compression. 18 Wistar rats were subjected to compression of the median nerve in the right elbow proximal region. Were randomly divided into G1 (untreated), G2 (NM for 1 minute), and G3 (NM for 3 minutes). For treatment, the animals were anesthetized and the right forelimb received mobilization adapted to humans, on alternated days, from the 3rd to the 13th day postoperatively (PO), totaling six days of therapy. Nociception was assessed by withdrawal threshold, and after euthanasia histomorphometric analysis of the median nerve was performed. The nociceptive evaluation showed in G2 and G3 delay in return to baseline. Histomorphometric analysis showed no significant differences in the variables analyzed. It is concluded that the NM was not effective in reducing nociceptive sensation and did not alter the course of nerve regeneration.

## 1. Introduction

Musculoskeletal diseases which are work related have reached epidemic proportions in recent decades. Among the major diseases caused by work, the carpal tunnel syndrome (CTS) can be highlighted, which is the most common peripheral neuropathy and is increasingly recognized as a cause of disability among workers [1].

The prevalence of CTS is estimated around 1% in the general population and is seen in 5% to 15% among workers in activities where there is use of repetitive flexion and extension of the wrist, intense gripping of hands, and incorrect wrist flexion when using heavy machinery or hand tools, which are considered risk factors for the syndrome development [2]. The results from median nerve compression at the radio-carpal and its classic symptoms are pain with nocturnal burning, hand numbness, and thenar atrophy. As a result, the performance of certain manual tasks may be limited or even impossible [3, 4].

It is estimated that every year one million American adults develop CTS, and approximately 450,000 surgeries are performed to release the carpal tunnel at a cost of $2 billion [5, 6]. Prime et al. [7] assert that, besides the direct economic consequences, one must take into account the financial implications and indirect load of absenteeism in the workplace. On average, workers affected by CTS remain about 27 days away from their professional duties.

Conservative treatment, which is reserved for patients whose symptoms are mild and slightly disabling [8], include immobilization of the wrist, physical therapy, acupuncture, drug therapy, and local steroids injections [9]. The surgical release of median nerve is reserved only for patients with severe or chronic symptoms. 75% of the patients treated show good results, but another 8% had worsening of symptoms after surgery [10, 11].

Physical therapy may be prescribed to all patients with CTS as the first therapeutic option because it considerably reduces the symptoms [2]. However, many of the physical therapy modalities, although they are often used, still do not have enough scientific background, because the results show only limited benefits in the short term [12].

Evidence supports that there is a positive correlation between the level of neural tension and severity of symptoms of CTS, suggesting that resources which reduce neural tension should be used [5]. The neural mobilization (MN) is a technique used to restore movement and neural elasticity, thus contributing to the resolution of the symptoms. Its goal is to improve and restore the neurodynamics and axoplasmic flow, restoring the homeostasis of the nervous tissue [13]. Despite being widely used among physiotherapists already, recent systematic review concluded that it is still not possible to say that the MN has positive impact on CTS [14].

Treatment of CTS is a point of great interest in health, and the use of injury models in animals is a very interesting option. Some works have already used experimental models to study the application of MN in peripheral nerve injuries [15–17], showing that it is possible to adapt the same technique used in humans to animals, given the similarity between the human upper limb and Wistar rat's forelimb. Further, Myers [18] states that nerve regeneration after ischemia and compression occurs through a very similar neuropathological response in humans as well as in rodents.

Considering that the scientific basis should be the guiding principle for the implementation of a reliable and efficient clinical practice, and although the evidence-based practice is now a reality in healthcare, there is an evident need for research that can support the conduct of physiotherapy, enabling the improvement of the quality of care provided to individuals with CTS.

This research proposes to investigate the efficacy of neural mobilization as a conservative treatment in Wistar rats' median nerve compression model, evaluating its effects on nociception and nerve histomorphometric characteristics and seeing if there are differences related to treatment time (1 to 3 minutes).

## 2. Materials and Methods

This research is experimental with quantitative approach. The project was conducted according to international ethics in animal experiments standards [19] and approved by the Animal Ethics and Practical Classes committee at Unioeste (1012/12).

We used 18 male Wistar rats, weighing 411.43 ± 31.75 g and 14 ± 2 weeks old, kept in 12 h photoperiod, 23 ± 1°C, with water and food ad libitum. The animals were randomly divided into three groups:

- G1 (*n* = 6): submitted to nerve compression and no mobilization (anesthesia only);
- G2 (*n* = 6): submitted to nerve compression and treated with neural mobilization for one minute;
- G3 (*n* = 6): submitted to nerve compression and treated with neural mobilization for three minutes.

### 2.1. Median Nerve Compression Protocol

The model used in order to accomplish the median nerve compression was the one presented by Chen et al. [20], which was based on the model of Bennett and Xie [21], performing a knot wired Catgut Chrome 4.0 by 4 points, with a distance of approximately 1 mm in the proximal region to the right elbow.

The aforementioned authors observed that the model generates painful symptoms and motor function is decreased, but, without producing self-mutilation, only member protection, which starts around the 3rd day increases nociception around the 7th day. To perform the surgical procedure, the animals were previously anesthetized with a solution of ketamine (50 mg/kg) and xylazine (10 mg/kg).

### 2.2. Median Nerve Mobilization Protocol

For the realization of the neural mobilization (G2 and G3), the animals were anesthetized and the treatment realized in the right forelimb were adjusted as indicated for treatment in humans [22]. The animals were placed in the supine position, with left lateral cervical flexion, shoulder girdle depression and mild abduction, and external rotation and supination, to the maximum extent possible in the elbow and wrist so that the resistance movement could be felt. Repeated oscillations were performed of wrist flexion: extension for one minute (G2) or three minutes (G3) in this position. The treatment period was from the 3rd to the 13th postoperative days (PO), on alternate days, totalizing six days of therapy. The animals of G1 were anesthetized and placed back in their boxes without undergoing the protocol.

### 2.3. Nociception Evaluation by Withdrawal Threshold

Nociception was assessed by the withdrawal threshold to mechanical stimulation. The equipment used to perform the test was the Von Frey digital analgesimeter (Insight). The equipment consists of a probe arm with a disposable polypropylene tip, with capacity from 0.1 to 1000 grams, connected to an amplifier box, measuring the pressure performed on the animal surface [23].

To facilitate the adaptation of animals to the instrument, in the three days prior to the injury, we performed a simulation of the evaluation. The animals were restrained manually, and Von Frey filament was applied in the region of nerve compression. The tip of the polypropylene filament was applied perpendicularly to the area, with gradual increase of pressure, and as soon as the animal withdrew the right forelimb, the test was interrupted to record the withdrawal threshold.

The test was performed before compression, so that basal values could be obtained (evaluation 1—EV1) and then 3 (EV2), 5 (EV3), 7 (EV4), 11 (EV5), 13 (EV6), and 14 (EV7) days after surgery, in order to observe the lesion evolution and the treatment modalities effect. The test was applied before the application of neural mobilization, except on the 14th postoperative day, because on this day the animals were not treated.

### 2.4. Histomorphometric Evaluation

Prior to animals decapitation, on the 14th postoperative day, the animal was anesthetized and the median nerve was dissected and removed 1 cm distal to the compression procedure. To establish a benchmark, 1 cm of the contralateral limb was also removed. The fragments of the nerves were put in 10% formalin, included in paraffin, and transverse sections with a 5 *μ*m thick were made. Subsequently, they were stained with hematoxylin and eosin.

A cut of each nerve was selected, and each slide was photographed. Using the 10x objective an image to estimate the nerve area was obtained. Using the 100x objective, images were captured of four visual fields, systematically located in the upper left, upper right, lower right, and lower left quadrants [24]. The images were analyzed with Image-Pro Plus 6.0. The quantity of axons in each visual field was counted, including the axons that were placed on the called “inclusion edges” (left and upper) and excluding the ones placed on the “exclusion edges” (right and bottom).

The measuring of the nerve fiber diameter (NFD), axon (AXD), and myelin sheath (MSD) was performed in 100 axons by nerve in order to obtain an equivalent number for comparisons between nerves. The ratio axon myelin and G Quotient were obtained, respectively, calculating MSD/AXD and AXD/NFD.

### 2.5. Data Analysis

Results were expressed and analyzed by descriptive and inferential statistics. We analyzed the data normality, using the Kolmogorov-Smirnov test. For functional evaluation, we used analysis of variance with repeated measures for intragroup analysis and one way for analysis between groups. For histomorphometric analysis we used the paired Student *t* test and one-way ANOVA for analysis within and between groups, respectively. In all cases the level of significance was set at 5%.

## 3. Results

### 3.1. Nociception Evaluation

Nociceptive evaluation results are shown in Table 1. All groups showed a significant increase to the values in the operated limb (right) comparing the results until EV4. However, in G2 and G3, this increase was still significant on the EV5.

### 3.2. Histomorphometric Analysis

Histomorphometric analysis results are presented in Table 2. In all variables, there are no significant differences.

## 4. Discussion

The functionality is the gold standard for evaluating the effectiveness of an intervention and allowing the implementation of their results to clinical practice [25]. Therefore, in this study, evaluation of nociception was used to monitor the functional recovery of the injured nerve. The three groups showed nociception that increased after median compression, as expected. The control group (G1) showed significant differences in nociception until the 7th day comparing with the baseline values. These data reaffirm the effectiveness of the model presented by Chen et al. [20] to produce painful symptoms and motor function reduction after nerve compression without, however, causing severe neuronal damage, given the fact that function returns to normal within a few days. A study by Silva et al. also found that hyperesthesia after median nerve compression was still significant until the 8th PO day [26].

The groups treated with neural mobilization (G2 and G3) also had increased nociceptive sensation after surgery, but, conversely to what was expected, there was longer persistence than in the control group. The altered values remained until day 11, suggesting slightly worse results compared to G1. Several factors may have contributed to these results.

The neural mobilization is a technique used in therapy, even though it is frequently based on researches with questionable methodology as the one of Oskay et al. [27]. Only subjects with mild to moderate CTS were selected and underwent an 8-week rehabilitation program including neural mobilization, cryotherapy, ultrasound, strengthening exercises, posture guidance, and ergonomic modifications. After the program the pain was reduced and there was improvement in the upper limb muscle strength, and after 12 months, the results were unchanged. However, it is important to mention that the neural mobilization was not applied alone.

It is also the case of the research of Pinar et al. [28], who applied the neural mobilization in CTS but associated with the use of the splint. One group of subjects underwent splint, and the other underwent the splint and neural mobilization. Both groups showed significant reduction in pain, but the grip strength showed a higher gain in the mobilized group. Akalin et al. [29] developed a similar study and showed a reduction in symptoms of CTS without, however, finding significant differences among group results.

Results similar to those of the present study were obtained by Bardak et al. [30], however, in humans. The authors randomized 111 patients into 3 groups: splint + local steroids injections (group 1), only neural mobilization (group 2), and both interventions (group 3). Only groups 1 and 3 achieved an improvement in symptoms, leading researchers to conclude that the neural mobilization alone was not effective in treating CTS.

Bertolini et al. [15] also used a peripheral nerve injury animal model, in which the authors applied the neural mobilization in sciatic nerve compression. They found that nociception went back to preinjury levels after 5 sessions, confirming the efficacy of treatment. However, the evaluation applied to assess functional parameters was the functional incapacitation test, which, along with other methodological differences, may explain the discrepancy between their results and those obtained in the present study. Based on these data, we also present the possibility that the unfavorable results in the present study were because of the nerve used.

An interesting point about the success of the neural mobilization in CTS is highlighted by McKeon and Yancosek [31]. In their review, they say that it is necessary to stratify subjects according to the severity of symptoms, as the neural mobilization is a nonsurgical treatment option and should be applied only to mild or moderate cases. Conservative treatment aims to delay or stop disease progression and subsequent surgical interventions, and thus for patients with severe CTS, surgery may be the only effective treatment option for relieving compression of the median nerve and reducing progression of the disease [31]. This assertion finds support in Oskay et al. [27] which followed this guidance and included in the rehabilitation program only mild or moderate cases. Even though the possibility of methodological bias has already been mentioned, the subjects treated with conservative rehabilitation program showed improvement.

In this experiment we observed the appearance of a more long-lasting hyperesthesia than expected in the treated groups. An important point to be considered is that the model used is based on an acute injury, while the CTS is usually chronic. The Chen et al. [20] research reveals that, following the first compression day, hypersensitivity can already be observed, extending up to the 14th day after injury. It is important to note that, at this stage of the inflammatory process, there is a number of proinflammatory mediators that are also algesic and may have been instrumental in the observed results. It is suggested that the type of compression used, plus the strain applied to median nerve by neural mobilization, may have caused acute inflammatory process, which would justify the excessive algic response observed.

Considering the initial phase of the inflammatory process, it is believed that better results could be obtained by starting the neural mobilization procedure at a later time after the injury, such as Santos et al. research [16]. The authors used the neural mobilization as a treatment for neuropathic pain induced by chronic compression of Wistar rat sciatic nerve. Unlike the present study, they have applied 10 sessions of neural mobilization, which just began 14 days after injury. Besides the decrease in symptoms, the author also noted favorable histologic results, showing that neural mobilization also favored the regeneration of sciatic nerve due to local increasing of nerve growth factor.

An additional factor deserves to be discussed. To implement the technique of neural mobilization, the recommendation to perform movements of passive extension of the wrist until some resistance to movement is felt was followed [22]. However, it is important to note that the animals were anesthetized, unlike what happens with treatment in humans, who can verbally mention any sign of discomfort, and therefore “control” of the amount of force applied by the therapist. In the anesthetized animal, this feedback is impossible to be obtained, and for this reason, the results of this research may have been limited and negatively influenced. Further studies could investigate the application of different parameters of passive force application on the animal limb or the use of different rates for oscillatory motions.

The histomorphometric variables analyzed showed no change in any of the groups studied, a fact that allows us to conclude that the injury was not serious; that is, the regeneration of nerve fibers was not affected by the treatment. The research of Neves [32] also emphasized the recovery of median nerve after crush injury in rats; however, the therapy used was a 4-week training program of balance and coordination. The objective was to determine the influence of these activities on the morphometric parameters of the damaged nerve (axonal area, axonal density, diameter myelinated fibers, axonal diameter, and thickness of the myelin sheath). Histological analysis showed that balance training and coordination accelerated the median nerve regeneration, since the results of axonal density and diameter were similar to those of the control group which was not injured.

In our study, despite the unfavorable results in nociceptive sensation, histomorphometric analysis showed no worsening of neural regeneration compared to the untreated group. The results were similar between groups, suggesting that the recovery was also similar. These data confirm the Pólvora [33] assertion, regarding the median nerve regeneration, which postulates that the morphometric data cannot be correlated with function.

Moreover, the hyperesthesia observed in the animals may have another explanation. It is known that nerve growth factor (NGF), which participates in the regeneration of peripheral nerve cells, is also an important mediator of pain [34]. Herzberg et al. [35] have suggested the NGF involvement in the hyperalgesia development after nerve compressions based on increased mRNA encoding NGF. In view of such information, it is suggested that the increase in NGF after injury, directed to the recovery of injured nerve, may have also caused increased nociceptive sensation. Future studies can investigate this question.

## 5. Conclusion

It is concluded that the neural mobilization was not effective as a conservative treatment in a nerve median compression model. There was no reduction of nociceptive sensation in the treated animals and, histologically, there were also no changes in nerve regeneration, delaying the proliferation of the number of axons in G3.

## Figures and Tables

**Table 1:** Results of the withdrawal threshold in forelimbs right (R) and left (L) (mean ± standard deviation in grams).

	G1		G2		G3	
	R	L	R	L	R	L
EV1	156.70 ± 56.72	148.10 ± 30.62	140.30 ± 19.23	123.30 ± 15.01	162.10 ± 39.91	157.00 ± 38.70
EV2	85.76 ± 28.22*	168.00 ± 35.83^•^	91.46 ± 3.85*	152.40 ± 27.48^•^	80.60 ± 29.91*	130.20 ± 34.58^•^
EV3	84.50 ± 17.71*	127.90 ± 15.54^•^	85.40 ± 25.23*	116.70 ± 5.33	98.34 ± 23.17*	122.30 ± 19.03^•^
EV4	89.70 ± 12.82*	137.70 ± 12.11^•^	86.06 ± 16.29*	109.50 ± 17.74^•^	76.20 ± 15.89*	134.00 ± 18.14^•^
EV5	89.26 ± 18.54	154.30 ± 84.50^•^	80.50 ± 17.50*	119.20 ± 13.54^•^	71.48 ± 22.21*	133.40 ± 17.30^•^
EV6	107.50 ± 12.20	147.10 ± 31.75	106.20 ± 23.62	137.50 ± 38.37^•^	117.70 ± 22.32	153.70 ± 22.71
EV7	100.00 ± 52.02	162.80 ± 14.71^•^	107.20 ± 23.10	152.40 ± 19.59	119.50 ± 26.94	145.30 ± 28.18

*Significant difference comparing with EV1 within the group. ^•^Significant difference when compared with the contralateral side.

**Table 2:** Results of histomorphometric analysis of the median nerve (mean ± standard deviation in *μ*m).

		Right	Left
Fiber diameter	G1	6.89 ± 0.78	7.62 ± 1.01
	G2	6.61 ± 1.49	7.37 ± 0.91
	G3	6.77 ± 1.35	7.53 ± 0.67

Axon diameter	G1	2.21 ± 0.25	2.47 ± 0.23
	G2	1.95 ± 0.59	2.16 ± 0.33
	G3	2.14 ± 0.42	1.99 ± 0.31

Myelin sheath diameter	G1	2.34 ± 0.27	2.54 ± 0.38
	G2	2.33 ± 0.47	2.61 ± 0.29
	G3	2.32 ± 0.49	2.77 ± 0.19

Myelin/axon	G1	1.06 ± 0.04	1.07 ± 0.08
	G2	1.27 ± 0.31	1.21 ± 0.07
	G3	1.09 ± 0.16	1.41 ± 0.17

Quotient G	G1	0.32 ± 0.01	0.33 ± 0.02
	G2	0.29 ± 0.04	0.29 ± 0.01
	G3	0.32 ± 0.03	0.26 ± 0.02
